# Supplementary material for: How compartmentalization and proliferation shape the splenic T cell repertoire during an immune response to a complex antigen in mice
Source: PLoS Comput Biol. 2026 Oct 14;22(10):e1014859. doi: 10.1371/journal.pcbi.1014859 (PMC13690269; doi:10.1371/journal.pcbi.1014859)
Supplement: Supplementary file 1 [file pcbi.1014859.s005.pdf]

Revision Report

PCOMPBIOL-D-25-02726

Dear editors,

We are grateful for the opportunity to address the reviewers comments and improve our work. We have revised our manuscript and supplemental materials, and have indicated changes in blue (for Figures that were updated, we have only marked the changed subfigure letters in blue, to distinguish from changes to the legend). We have included a point-by-point response to each issue raised below, where the original comments from the reviewers are marked in shaded blocks.

Kind regards,
The authors

Point-by-point responses

Reviewer 1

> The manuscript by Buytenhuijs et al. is a study that examines TCR repertoires within splenic compartments in unimmunized and immunized mice using a combination of histology, microdissection, RNA-sequencing, and down selection/population modeling.
>
> Overall, this study is well executed and convincingly shows the stochastic crossover of T cells from the T cell zone to the B cell zone in the unimmunized setting. In the context of immunization, the analysis here nicely identifies drivers of differences between compartments (i.e., local proliferation/selection). My enthusiasm is slightly dampened by the following major points.

Thank you, we greatly appreciate your feedback.

Major Points

1) Regarding qualitative nature of TCR repertoires remaining stable across compartments following immunization. Although this study was performed with a model complex immunogen (sheep red blood cells), this conclusion is somewhat strong given the data provided. A second immunization arm would be helpful to help establish the point that the TCR repertoire qualitatively does not shift despite clonal expansion/migration. It is possible that this conclusion is true for immunization with sheep red blood cells but could differ in the context of another (similarly complex) immunogen. If it is not feasible to add another immunogen to this study, I suggest tuning down this conclusion.

Unfortunately, we are not able to add a second immunization arm. We have made the following changes to the manuscript to soften our conclusion:

Under “Discussion”:

“Here, we have used SRBC as our model antigen. It is possible that TCR-R profiles in immune responses with different antigens are structurally different and cannot be explained through differences in compartment size and proliferation.”

We have also updated the title of our manuscript to indicate that we are considering a complex antigen:

“How compartmentalization and proliferation shape the splenic T cell repertoire during an immune response to a complex antigen in mice”

2) In Figure 5 is there a reason why clusters are defined by a hamming distance of 1? Do the graphs and interpretation change if a cluster is defined by a hamming distance of 0 or 2? If they do not, it would be helpful to include that data as part of the supplement.

We are examining clusters of sequences based on sequence similarity to identify related TCRs, since TCRs responding to the same antigen often have similar sequences. For this, it makes sense to define these groupings with a very small distance. Sequences with a Hamming distance of 0 are the same and therefore this will not create any clusters. A hamming distance of 1 is therefore the smallest distance for grouping sequences. It is possible to use a hamming distance of 2, allowing up to 2 amino acids to be different. This results in larger clusters than with a distance of 1, as shown in the figure below. We have not included this in the manuscript since we do not think this is a relevant addition, but are happy to include this if the reviewers deem it necessary.

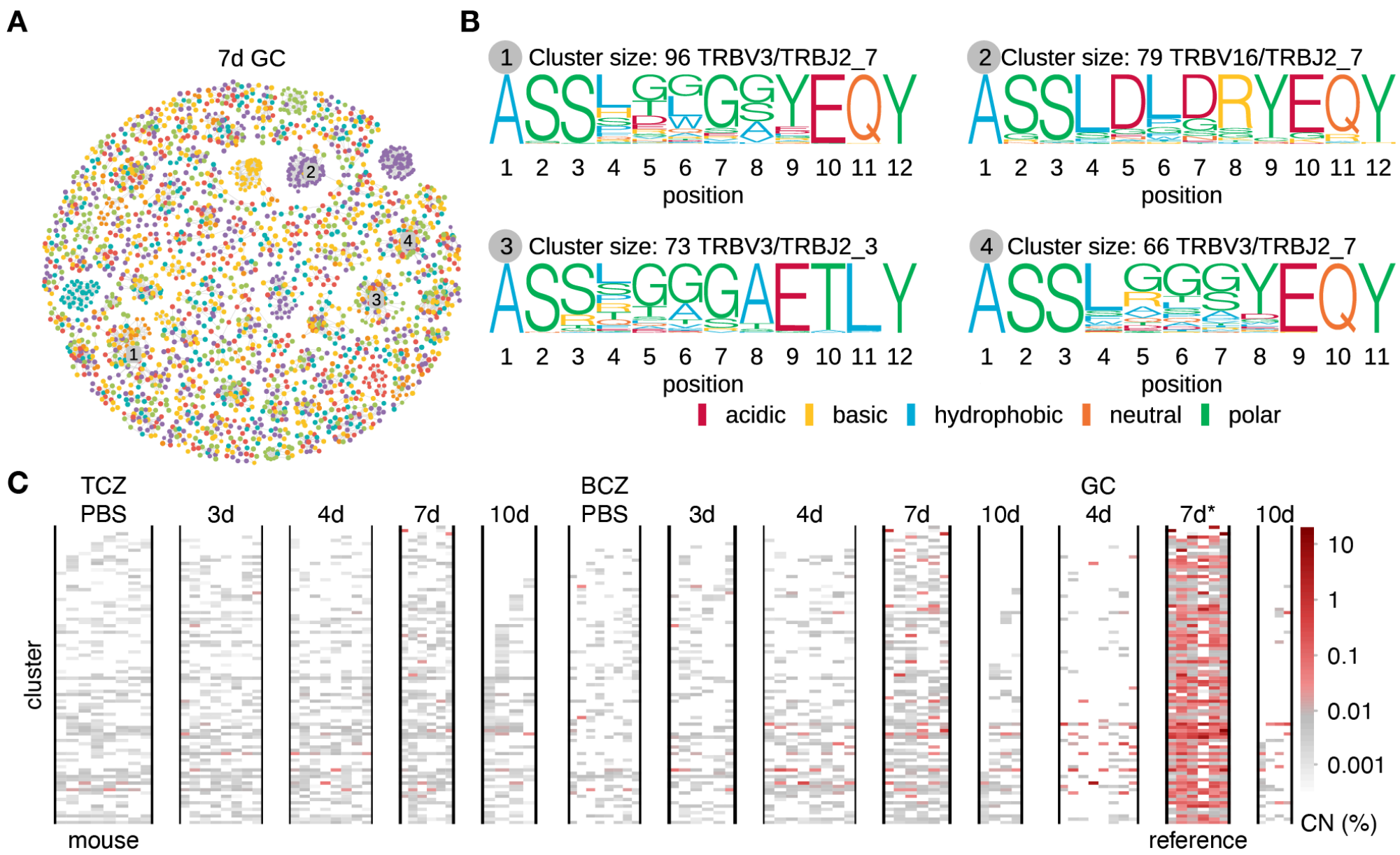

Revision Figure R1. Recreation of Figure 5 with Hamming distance of 2. Clusters are similar but larger. Cluster 1 corresponds to (has the same V/J segments as) cluster 2 of Figure 5, cluster 3 corresponds to cluster 1 of Figure 5, cluster 4 corresponds to cluster 2 of Figure 5. A substantial portion of clonotypes from GC clusters could be detected in early stages, and copy numbers increase over time.

We have made a small change in the manuscript:

Under "Results", section "The GC TCRR contains clusters of similar sequences that can be traced back to the early immune response":

"Sequences were connected if they shared the same V gene and J gene and their CDR3β junction sequence differed in at most one amino acid (Hamming distance of 1)."

Minor Point

1) It would be helpful to include as part of the discussion, expected TCR repertoire behavior of lymph nodes, as it is another secondary lymphoid organ. Given the relative difference in T cell abundance within a lymph node versus a spleen, I would assume that interpretation of the splenic data should not be directly applied to the much smaller lymph node.

We have added the following to our manuscript:

Under “Discussion”:

“Furthermore, we have not studied the immune response and T- and B-cell migration in the lymph nodes. Given the similar microanatomic structure, we assume that observed differences in TCRR in the TCZ, BCZ, and GC of the lymph nodes can also be explained by size and proliferation, but further research could verify this.”

Reviewer 2

> In this article, the authors investigate the T cell repertoire in different splenic compartments of mice without and after immunization. Comparing TCR repertoires in the T cell zone, B cell zone and germinal centres, and analysing the data by using a mathematical model for cell proliferation dynamics, they find that substantial differences in TCR composition observed between compartments could be explained if compartment size and proliferation dynamics were accounted for. Based on these observations they conclude that compartment-wise differences in splenic TCR-repertoires are not determined by unique TCR features but rather shaped by the compartment size and proliferation dynamics.
>
> Overall, the manuscript covers an interesting and relevant topic to understand T cell response dynamics by combining experimental data and mathematical modelling. In general, the manuscript is well written and structured. However, some of the used mathematical analyses should be explained in more detail, as it is sometimes difficult to follow the results and conclusions. There are some points which would require some additional explanations and clarifications:

Thank you, we greatly appreciate your feedback.

Major points

1) A large part of the analyses and conclusions relies on the application of a Galton-Watson branching process to the data. However, the process and, thus, the model itself is only insufficiently described in the methods section. For a reader not familiar with the Galton-Watson process it is not clear if for each clone a random proliferation rate is considered, which is constant over time, or if this proliferation rate varies with each division. In addition, details on the fitting procedure are missing. Which algorithms were used to adjust/minimize the difference between the observed and simulated dynamics? What parameters are actually estimated/fitted, and what are the estimated values for each of the different compartments and time points? Judging from the text (lines 216-219) and the figures, it seems that each day is fitted separately, but this is not totally clear. If each day was fitted separately, did the authors also consider to simultaneously fit the clone size distributions of all days, and why was this neglected?

The proliferation rate is constant over time. For a range of proliferation rates, the Galton-Watson process is run and the subsequent dataset is saved. For each dataset, the KL divergence is calculated with respect to the BCZ or GC. The optimal proliferation rate, i.e. that proliferation rate that best explains the data, is the rate for which the KL divergence is the lowest. We have clarified this in the methods.

Under "Methods", section "TCR-R data analysis", subsection "Cell expansion modelling":

"The process begins with the initial downsampled dataset in the first evolution step. During this step, each clonotype in the dataset attempts to proliferate, succeeding with a probability x. When proliferation is successful, the clonotype divides into two daughter cells. In subsequent evolution steps, the newly generated daughter cells from the previous step undergo the same proliferation process with the same proliferation probability. This iterative cycle continues until no new cells are produced in a given evolution step, marking the conclusion of the proliferation cycle.

Once the cycle is completed, the total number of clonotypes generated is used to calculate a new copy number. In the case of positive expansion probabilities (x>0), the original copy numbers for each sequence are multiplied by the number of offspring produced, modeling a situation where proliferation is higher in the smaller compartment. For negative expansion (x<0), the original copy numbers are divided by the number of offspring produced. The negative probability models a situation where the expansion rate is higher in the larger compartment than in the smaller compartment.

For modelling of the BCZ with the TCZ, we ranged the proliferation probability between 0.35 and 0.35. For modelling of the GC with the BCZ, we observed that larger values were a better fit and therefore used a larger range of 0.475 to 0.475. We compared the clonotype distributions of the TCRRs for each proliferation probability to the TCRRs of the BCZ and GC."

Todo: justification of separation of days

2) In line with comment 1, it would be good to show the actual parameter estimates of the proliferation dynamics for each of the different compartments (and time points?), e.g. as a table including also confidence intervals of estimates. This would help to judge potential differences between compartments, which are only briefly mentioned in the text by stating particular numbers (e.g. line 242-243). A more systematic overview of the estimated parameters would be helpful to judge the postulated differences and inferred conclusions.

We have added the proliferation rate parameter that was used to Figures 2D and 3G. This is the only estimated parameter. The estimation is shown in Figures 2C and 3F. The parameter per day is also visualized separately in Supplementary Figure 2.

3) For several figures (e.g. Fig2 A,C,D ; Fig. 3E,G) it is not clear if the dashed lines actually correspond to model predictions or are simple spline-functions used to interpolate the dynamics of the measured data. If these are model predictions it looks a bit like overfitting as they are matching all data points very well. If these are not model predictions, I think figures showing model predictions vs. actual data are missing?

The dashed lines are indeed spline-functions. For Figure 2, the real data is from TCZ in black and BCZ in blue. We have called the simulations dsT and dsTx, which are in grey. For Figure 3, the real data is from BCZ in blue and GC in green. We have called the simulations dsB and dsBx, in grey. We compare the dsT/BCZ, dsTx/BCZ, dsB/GC, and dsBX/GC with the KL divergence and with the geometric mean. We used a mixture of solid, dashed and dotted lines for this, we have now converted everything to dashed lines.

4) I also assume that each compartment is analysed separately by applying a Galton-Watson process to the data. In this regard, increased proliferation rates in smaller compartments (e.g. B-cell zone) are interpreted as potential migration dynamics from a larger source compartment (e.g. T-cell zone). However, in line with the comment (1) above, wouldn't it be possible to also fit a branching process model that additionally accounts for the migration of cells from the T cell zone to the B cell zone?

We agree that it would be great to include migration as a variable. However, with this data, we are unable to include migration, since various combinations of migration rate and proliferation rate could explain the data. We have extended the discussion on this part.

Under “Discussion”:

“Additionally, our model cannot distinguish whether T cells initially proliferated in one compartment and later migrated to another or if proliferation occurred exclusively within a single compartment, as multiple combinations of proliferation and migration parameters could explain the data, limiting our ability to resolve the exact sequence of these events.”

5) Looking at the clone size distributions for the different compartments and time points it looks a bit as if they slightly differ from other clone size distributions published previously, i.e., indicating few large clones and a lot of small clones (e.g. Gerlach et al. Science 2013). While I might also be misled by differences in the presentation, the authors might want to discuss their findings also in comparison to previous results on T cell clone size distributions.

We are observing the same clone size distributions for the different compartments as Gerlach at al., 2013, with few large clones and a lot of small clones. However, we used a logarithmic binning, since this best described the data distribution, as added in Supplementary Figure 1. If we plot the reads without binning, we see the same distribution as the mentioned paper. It’s important to note that we are examining all clones, and not just the antigen specific clones, like Gerlach et al.

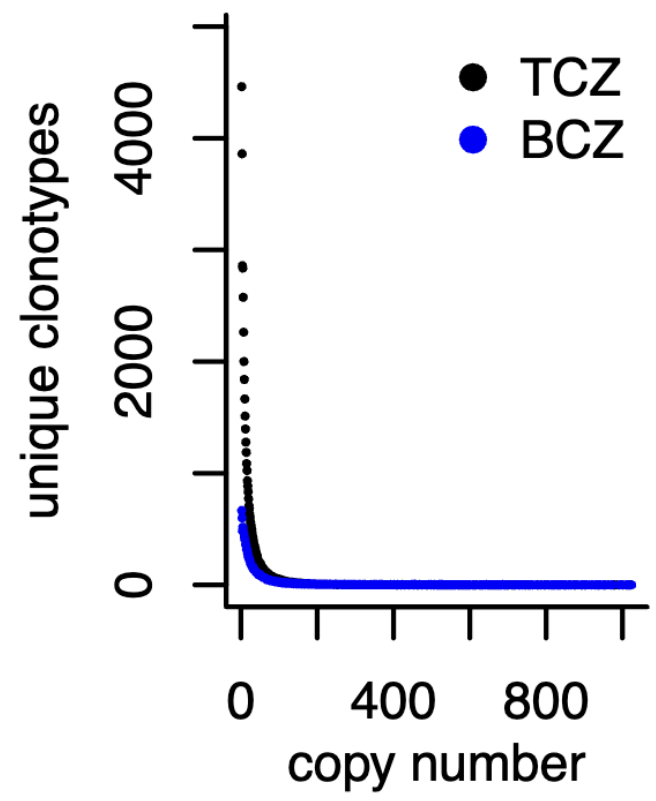

Revision Figure R2. Unbinned clone size distribution of TCZ and BCZ in PBS, with few large clones and many small clones. Figure 1F shows the same data with logarithmic binning.

Minor points

1) As the authors study and compare the diversity of the TCR repertoire at different sites, and also between different mice (e.g. Figure 5), could the authors argue why standard measurements of diversity and similarity (e.g. Simpson-Diversity Index, Morisita-Horn-Index, etc) were not used or useful to characterise the samples, as they could also account for different sample sizes (e.g. Venturi et al J. Immunol. Methods 2007 and 2008)?

In this manuscript, we are examining the clonotype counts, and shifts in the counts distributions throughout immunization. We are not looking at the diversity of the repertoire itself. This could be an extension of this work. We have added the following to the manuscript.

Under “Discussion”:

“Therefore, despite highly dynamic T cell expansion and migration, the qualitative properties of the TCRRs as a whole appear to have been preserved. Further research can focus on sequences and diversity of the repertoire in different zones throughout the immune response.”

2) The sketch in Figure 2B could be improved by additionally incorporating the copy numbers in each cell after each division. In addition, I think there is a typo as 8x4 should be 32 and not 24? I am also not sure how to interpret the arrows indicating the geometric mean in panel A, C, and D. It is not clear if they clearly point to the geometric mean on the x-axis or are sometimes scattered for graphical clarity. Maybe a different representation should be considered, as e.g. vertical dashed lines?

We have updated Figure 2B. Since each dividing cell would keep the same count and it’s the overall number of cells that changes through division, we have tried to more explicitly indicate how the copy number changes after proliferation.

For the geometric mean in Figure 2A/D, the scattering for overlapping arrows was only in the y-direction, but we agree that this was not clear. We have aligned all arrows on the same height.

3) Figure 3E,F,G: I would suggest to align the y-axis label around both plots of each panel (same applies to other figures, such as Fig.2). I had some difficulties to directly see the relationship between the different plots. In addition, the y-axis labelling in panel F could be turned to clearly see the "0" of the upper plot.

We have aligned the y-axis in Figure 2 and 3 as suggested. This way, the shift in distribution is less visible since the x-axis do not align anymore, but it is visible in Supplementary Figure S2. We have also flipped the y-axis labelling in panel 3F.

4) Figure 5 legend: Potential typo/missing word? "Cluster of T cells in GC mice ..." maybe "Cluster of T cells in GC of mice ..."?

Thank you for pointing this out, we have fixed it.

Reviewer 3

The authors investigate how TCR repertoires vary across splenic compartments during an immune response by combining sequencing of CDR3β regions with computational downsampling and proliferation modeling. While the approach is conceptually innovative and addresses an important biomedical challenge, the manuscript requires major revision due to insufficient methodological clarity, validation gaps, and limited biological interpretation in key sections.

Thank you, we greatly appreciate your feedback.

Major Comments

1) Downsampling strategy
In this study, the authors compared TCR repertoires between splenic compartments by downsampling the larger TCZ dataset to match the BCZ dataset. This was done using sampling weighted by the $\log_2$ copy number of each clonotype to account for abundance differences. It would be helpful if the authors briefly justified the choice of $\log_2$ weighting during the downsampling process. Because this transformation compresses differences between highly expanded clones, it may influence the resulting clone-size distributions. A short explanation or sensitivity test comparing alternative sampling approaches (e.g., linear weighting or proportional sampling based on raw counts) would strengthen confidence that the results are not sensitive to the specific downsampling strategy used.

We compared different weighting of probabilities in downsampling, and found that $\log_2$ of copy number was the best fit. We have added a supplementary figure showing different downsampling weightings (Supplementary Figure S1), and have added the following 2 changes in the manuscript:

Under “Methods”, section “TCR-R data analysis”, subsection “Downsampling”:

“CDR3β amino acid sequences were sampled in batches of 50, with replacement. We examined different weighting of the probability of selecting sequences, including unweighted (random), and weighted by the copy number, base-2 (log_2) of the copy number, and the generation probability P_{gen}, calculated with OLGA,[22] and compared the geometric mean of each method with that of the BCZ. The closest method was the log_2 of the copy number, with which we proceeded. This weighting ensured that more abundant sequences were more likely to be included in the sample. This iterative sampling continued until the number of unique amino acid sequences in the TCZ sample matched that in the BCZ, within a tolerance of ±25 sequences.”

Under “Results”, section “Similar CDR3β TCR-Rs in the T cell zone (TCZ) and B cell zone (BCZ) in the absence”:

“To determine to what extent these apparent differences were merely driven by the different numbers of T cells in the samples, we used a subsampling approach where we randomly selected the same number of clonotypes found in a BCZ sample from the TCZ sample for each animal (see Methods, Figure 1E). We weighted probabilities of sequence selection by the (log2) of the copy number after comparing various methods (Supplementary Figure S1).”

2) Use of KL divergence for distribution comparison
To quantify differences between clonotype size distributions across compartments, the authors used Kullback–Leibler (KL) divergence as the primary metric for comparing distributions. This metric is used throughout the manuscript to identify the best-fitting proliferation parameters in the modeling framework. While KL divergence is appropriate for distribution comparison, it is asymmetric and can be sensitive to low-frequency bins, which are common in TCR repertoire datasets. The authors might consider briefly confirming that their conclusions remain consistent when using an additional distribution similarity metric (e.g., Jensen–Shannon divergence or Kolmogorov–Smirnov statistic). This would demonstrate that the observed trends are robust to the choice of distance metric.

We have included a new supplemental figure (Supplementary Figure S2) that examines different similarity statistics (Kullback–Leibler divergence, Jensen–Shannon divergence, Kolmogorov–Smirnov statistic, and mean squared error). We observe the same trends for all statistics, specifically, a negative expansion rate on day 3 (thus a higher proliferation rate in the TCZ than BCZ), and a high positive proliferation rate on day 4 (thus a higher proliferation rate in the BCZ than TCZ).

We have added the following sentences to our manuscript:

Under “Methods”, section “TCR-R data analysis”, subsection “Statistical analysis”:

“The distributions of unique clonotypes are compared using KullbackLeibler (KL) divergence. The KL divergence is a statistical measure that quantifies the distance between two distributions, where zero means two distributions are equal. To verify that

our interpretation is robust to the choice of metrics, we used Mean Squared Error, Jensen-Shannon divergence, and Kolmogorov-Smirnov distance."

Under "Results", section "The differences between the TCZ and BCZ during an immune response are consistent with increased proliferation in the BCZ:

"Using the best fitting proliferation rates, the dsTx distribution closely resembled that of the BCZ (Figure 2D). We verified that similar expansion rates were found using alternative metrics (Supplementary Figure 2)."

3) Removal of singleton sequences
The authors removed sequences with only one mapped read prior to analysis in order to reduce the influence of potential sequencing errors. This filtering step is commonly used in immune repertoire analyses. However, removing singleton reads can substantially alter the representation of rare clonotypes, which contribute to overall repertoire diversity. It would be useful if the authors briefly reported the proportion of sequences removed by this filtering step or commented on whether this removal significantly affects diversity estimates or clone-size distributions. Even a short clarification would help readers evaluate the potential impact of this preprocessing decision.

We added information on sequence removal in the manuscript.

Under "Methods", section "TCR-R data analysis", subsection "Statistical analysis":

"Because they have the highest risk of containing sequencing errors, sequences with only one mapped read were removed from all analyses. Furthermore, sequences with a stop codon or uncertainty (or ~) and without a P_{gen} were removed. In total, 31.6% of sequences were removed, with a higher percentage in the GC (59.9%) than in the TCZ (29.9%) and BCZ (33.3%)."*

Minor Comments

1) In the Methods section, the phrase "during an the immune response" appears to contain a grammatical error. It would read more clearly as "during the immune response." A careful proofreading of the manuscript may help identify similar minor typographical issues.

Thank you for pointing this out, we have fixed this issue, and several others.

2) The manuscript alternates between the terms TCR-R and TCR repertoire. While both refer to the same concept in my understanding, it may improve readability if the terminology is standardized throughout the manuscript or clearly defined once and used consistently thereafter.

Thank you, we have standardized the terminology.

3) The manuscript uses several formats to describe time points (e.g., “3d”, “3 days p.i.”, and “3 days post-immunization”). For clarity, the authors may consider using a single consistent notation throughout the text and figures.

Thank you, we’ve improved our consistency. We do introduce the abbreviation again in figure legends, since these should stand on their own.

4) In the Introduction, the phrase describing TCR diversity (“estimated to range from 10^{15} and 10^{20} distinct sequences”) would read more clearly as “estimated to range from 10^{15} to 10^{20} distinct sequences.” This appears to be a small wording issue.

Thank you for pointing this out, we have fixed this issue.
