# Supplementary material for: How compartmentalization and proliferation shape the splenic T cell repertoire during an immune response to a complex antigen in mice
Source: PLoS Comput Biol. 2026 Oct 14;22(10):e1014859. doi: 10.1371/journal.pcbi.1014859 (PMC13690269; doi:10.1371/journal.pcbi.1014859)
Supplement: Peer review history

# Peer review: Decision Letter 0

**DOI:** 10.1371/journal.pcbi.1014859.r001

**Authors:** James Faeder, Philipp Altrock

12 Mar 2026

How compartmentalization and proliferation shape the splenic T cell repertoire during an immune response in mice

PLOS Computational Biology

Dear Dr. Textor,

Thank you for submitting your manuscript to PLOS Computational Biology. After careful consideration, we feel that it has merit but does not fully meet PLOS Computational Biology's publication criteria as it currently stands. Therefore, we invite you to submit a revised version of the manuscript that addresses the points raised during the review process.

Please submit your revised manuscript by May 12 2026 11:59PM. If you will need more time than this to complete your revisions, please reply to this message or contact the journal office at ploscompbiol@plos.org. When you're ready to submit your revision, log on to https://www.editorialmanager.com/pcompbiol/ and select the 'Submissions Needing Revision' folder to locate your manuscript file.

Please include the following items when submitting your revised manuscript:

* A letter that responds to each point raised by the editor and reviewer(s). You should upload this letter as a separate file labeled 'Response to Reviewers'. This file does not need to include responses to formatting updates and technical items listed in the 'Journal Requirements' section below.

* A marked-up copy of your manuscript that highlights changes made to the original version. You should upload this as a separate file labeled 'Revised Manuscript with Track Changes'.

* An unmarked version of your revised paper without tracked changes. You should upload this as a separate file labeled 'Manuscript'.

If you would like to make changes to your financial disclosure, competing interests statement, or data availability statement, please make these updates within the submission form at the time of resubmission. Guidelines for resubmitting your figure files are available below the reviewer comments at the end of this letter

We look forward to receiving your revised manuscript.

Kind regards,

Philipp Martin Altrock, Ph.D.

Academic Editor

PLOS Computational Biology

James Faeder

Section Editor

PLOS Computational Biology

**Journal Requirements:**

1) Please ensure that the CRediT author contributions listed for every co-author are completed accurately and in full.

At this stage, the following Authors/Authors require contributions: Franka Buytenhuijs, Cornelia Tune, Andrea Schampel, Lisa-Kristin Schierloh, René Pagel, Jürgen Westermann, and Johannes Textor. Please ensure that the full contributions of each author are acknowledged in the "Add/Edit/Remove Authors" section of our submission form.

The list of CRediT author contributions may be found here: https://journals.plos.org/ploscompbiol/s/authorship#loc-author-contributions

2) We ask that a manuscript source file is provided at Revision. Please upload your manuscript file as a .doc, .docx, .rtf or .tex. If you are providing a .tex file, please upload it under the item type u2018LaTeX Source Fileu2019 and leave your .pdf version as the item type u2018Manuscriptu2019.

3) Please provide an Author Summary. This should appear in your manuscript between the Abstract (if applicable) and the Introduction, and should be 150-200 words long. The aim should be to make your findings accessible to a wide audience that includes both scientists and non-scientists. Sample summaries can be found on our website under Submission Guidelines:

https://journals.plos.org/ploscompbiol/s/submission-guidelines#loc-parts-of-a-submission

4) Please upload all main figures as separate Figure files in .tif or .eps format. For more information about how to convert and format your figure files please see our guidelines:

https://journals.plos.org/ploscompbiol/s/figures

5) We notice that your supplementary Figure is included in the manuscript file. Please remove it and upload it with the file type 'Supporting Information'. Please ensure that each Supporting Information file has a legend listed in the manuscript after the references list.

6) Please amend your detailed Financial Disclosure statement. This is published with the article. It must therefore be completed in full sentences and contain the exact wording you wish to be published.

1) State what role the funders took in the study. If the funders had no role in your study, please state: "The funders had no role in study design, data collection and analysis, decision to publish, or preparation of the manuscript."

7) Please ensure that the funders and grant numbers match between the Financial Disclosure field and the Funding Information tab in your submission form. Note that the funders must be provided in the same order in both places as well.

Note: If the reviewer comments include a recommendation to cite specific previously published works, please review and evaluate these publications to determine whether they are relevant and should be cited. There is no requirement to cite these works unless the editor has indicated otherwise.

**Reviewers' comments:**

Reviewer's Responses to Questions

Reviewer #1: The manuscript by Buytenhuijs et al. is a study that examines TCR repertoires within splenic compartments in unimmunized and immunized mice using a combination of histology, microdissection, RNA-sequencing, and down selection/population modeling.

Overall, this study is well executed and convincingly shows the stochastic crossover of T cells from the T cell zone to the B cell zone in the unimmunized setting. In the context of immunization, the analysis here nicely identifies drivers of differences between compartments (i.e., local proliferation/selection). My enthusiasm is slightly dampened by the following major points.

Major Points:

1) Regarding qualitative nature of TCR repertoires remaining stable across compartments following immunization. Although this study was performed with a model complex immunogen (sheep red blood cells), this conclusion is somewhat strong given the data provided. A second immunization arm would be helpful to help establish the point that the TCR repertoire qualitatively does not shift despite clonal expansion/migration. It is possible that this conclusion is true for immunization with sheep red blood cells but could differ in the context of another (similarly complex) immunogen. If it is not feasible to add another immunogen to this study, I suggest tuning down this conclusion.

2) In Figure 5 is there a reason why clusters are defined by a hamming distance of 1? Do the graphs and interpretation change if a cluster is defined by a hamming distance of 0 or 2? If they do not, it would be helpful to include that data as part of the supplement.

Minor Point:

1) It would be helpful to include as part of the discussion, expected TCR repertoire behavior of lymph nodes, as it is another secondary lymphoid organ. Given the relative difference in T cell abundance within a lymph node versus a spleen, I would assume that interpretation of the splenic data should not be directly applied to the much smaller lymph node.

Reviewer #2: In this article, the authors investigate the T cell repertoire in different splenic compartments of mice without and after immunization. Comparing TCR repertoires in the T cell zone, B cell zone and germinal centres, and analysing the data by using a mathematical model for cell proliferation dynamics, they find that substantial differences in TCR composition observed between compartments could be explained if compartment size and proliferation dynamics were accounted for. Based on these observations they conclude that compartment-wise differences in splenic TCR-repertoires are not determined by unique TCR features but rather shaped by the compartment size and proliferation dynamics.

Overall, the manuscript covers an interesting and relevant topic to understand T cell response dynamics by combining experimental data and mathematical modelling. In general, the manuscript is well written and structured. However, some of the used mathematical analyses should be explained in more detail, as it is sometimes difficult to follow the results and conclusions. There are some points which would require some additional explanations and clarifications:

# Major points:

(1.) A large part of the analyses and conclusions relies on the application of a Galton-Watson branching process to the data. However, the process and, thus, the model itself is only insufficiently described in the methods section. For a reader not familiar with the Galton-Watson process it is not clear if for each clone a random proliferation rate is considered, which is constant over time, or if this proliferation rate varies with each division. In addition, details on the fitting procedure are missing. Which algorithms were used to adjust/minimize the difference between the observed and simulated dynamics? What parameters are actually estimated/fitted, and what are the estimated values for each of the different compartments and time points? Judging from the text (lines 216-219) and the figures, it seems that each day is fitted separately, but this is not totally clear. If each day was fitted separately, did the authors also consider to simultaneously fit the clone size distributions of all days, and why was this neglected?

(2.) In line with comment 1, it would be good to show the actual parameter estimates of the proliferation dynamics for each of the different compartments (and time points?), e.g. as a table including also confidence intervals of estimates. This would help to judge potential differences between compartments, which are only briefly mentioned in the text by stating particular numbers (e.g. line 242-243). A more systematic overview of the estimated parameters would be helpful to judge the postulated differences and inferred conclusions.

(3.) For several figures (e.g. Fig2 A,C,D ; Fig. 3E,G) it is not clear if the dashed lines actually correspond to model predictions or are simple spline-functions used to interpolate the dynamics of the measured data. If these are model predictions it looks a bit like overfitting as they are matching all data points very well. If these are not model predictions, I think figures showing model predictions vs. actual data are missing?

(4.) I also assume that each compartment is analysed separately by applying a Galton-Watson process to the data. In this regard, increased proliferation rates in smaller compartments (e.g. B-cell zone) are interpreted as potential migration dynamics from a larger source compartment (e.g. T-cell zone). However, in line with the comment (1) above, wouldn't it be possible to also fit a branching process model that additionally accounts for the migration of cells from the T cell zone to the B cell zone?

(5.) Looking at the clone size distributions for the different compartments and time points it looks a bit as if they slightly differ from other clone size distributions published previously, i.e., indicating few large clones and a lot of small clones (e.g. Gerlach et al. Science 2013). While I might also be misled by differences in the presentation, the authors might want to discuss their findings also in comparison to previous results on T cell clone size distributions.

# Minor points:

- As the authors study and compare the diversity of the TCR repertoire at different sites, and also between different mice (e.g. Figure 5), could the authors argue why standard measurements of diversity and similarity (e.g. Simpson-Diversity Index, Morisita-Horn-Index, etc) were not used or useful to characterise the samples, as they could also account for different sample sizes (e.g. Venturi et al J. Immunol. Methods 2007 and 2008)?

- The sketch in Figure 2B could be improved by additionally incorporating the copy numbers in each cell after each division. In addition, I think there is a typo as 8x4 should be 32 and not 24? I am also not sure how to interpret the arrows indicating the geometric mean in panel A, C, and D. It is not clear if they clearly point to the geometric mean on the x-axis or are sometimes scattered for graphical clarity. Maybe a different representation should be considered, as e.g. vertical dashed lines?

- Figure 3E,F,G: I would suggest to align the y-axis label around both plots of each panel (same applies to other figures, such as Fig.2). I had some difficulties to directly see the relationship between the different plots. In addition, the y-axis labelling in panel F could be turned to clearly see the "0" of the upper plot.

- Figure 5 legend: Potential typo/missing word? "Cluster of T cells in GC mice ..." maybe "Cluster of T cells in GC of mice ..."?

Reviewer #3: Reviewer Comments

Title: How compartmentalization and proliferation shape the splenic T cell repertoire during an immune response in mice

Manuscript id: PCOMPBIOL-D-25-02726

Overall Recommendation: Major Revision

The authors investigate how TCR repertoires vary across splenic compartments during an immune response by combining sequencing of CDR3β regions with computational downsampling and proliferation modeling. While the approach is conceptually innovative and addresses an important biomedical challenge, the manuscript requires major revision due to insufficient methodological clarity, validation gaps, and limited biological interpretation in key sections.

Major Comments:

1. Downsampling strategy

In this study, the authors compared TCR repertoires between splenic compartments by downsampling the larger TCZ dataset to match the BCZ dataset. This was done using sampling weighted by the log₂ copy number of each clonotype to account for abundance differences. It would be helpful if the authors briefly justified the choice of log₂ weighting during the downsampling process. Because this transformation compresses differences between highly expanded clones, it may influence the resulting clone-size distributions. A short explanation or sensitivity test comparing alternative sampling approaches (e.g., linear weighting or proportional sampling based on raw counts) would strengthen confidence that the results are not sensitive to the specific downsampling strategy used.

2. Use of KL divergence for distribution comparison

To quantify differences between clonotype size distributions across compartments, the authors used Kullback–Leibler (KL) divergence as the primary metric for comparing distributions. This metric is used throughout the manuscript to identify the best-fitting proliferation parameters in the modeling framework. While KL divergence is appropriate for distribution comparison, it is asymmetric and can be sensitive to low-frequency bins, which are common in TCR repertoire datasets. The authors might consider briefly confirming that their conclusions remain consistent when using an additional distribution similarity metric (e.g., Jensen–Shannon divergence or Kolmogorov–Smirnov statistic). This would demonstrate that the observed trends are robust to the choice of distance metric.

3. Removal of singleton sequences

The authors removed sequences with only one mapped read prior to analysis in order to reduce the influence of potential sequencing errors. This filtering step is commonly used in immune repertoire analyses. However, removing singleton reads can substantially alter the representation of rare clonotypes, which contribute to overall repertoire diversity. It would be useful if the authors briefly reported the proportion of sequences removed by this filtering step or commented on whether this removal significantly affects diversity estimates or clone-size distributions. Even a short clarification would help readers evaluate the potential impact of this preprocessing decision.

Minor Comments:

1. In the Methods section, the phrase “during an the immune response” appears to contain a grammatical error. It would read more clearly as “during the immune response.” A careful proofreading of the manuscript may help identify similar minor typographical issues.

2. The manuscript alternates between the terms TCR-R and TCR repertoire. While both refer to the same concept in my understanding, it may improve readability if the terminology is standardized throughout the manuscript or clearly defined once and used consistently thereafter.

3. The manuscript uses several formats to describe time points (e.g., “3d”, “3 days p.i.”, and “3 days post-immunization”). For clarity, the authors may consider using a single consistent notation throughout the text and figures.

4. In the Introduction, the phrase describing TCR diversity (“estimated to range from 10¹⁵ and 10²⁰ distinct sequences”) would read more clearly as “estimated to range from 10¹⁵ to 10²⁰ distinct sequences.” This appears to be a small wording issue.

**********

**Have the authors made all data and (if applicable) computational code underlying the findings in their manuscript fully available?**

The PLOS Data policy requires authors to make all data and code underlying the findings described in their manuscript fully available without restriction, with rare exception (please refer to the Data Availability Statement in the manuscript PDF file). The data and code should be provided as part of the manuscript or its supporting information, or deposited to a public repository. For example, in addition to summary statistics, the data points behind means, medians and variance measures should be available. If there are restrictions on publicly sharing data or code —e.g. participant privacy or use of data from a third party—those must be specified.

Reviewer #1: Yes

Reviewer #2: Yes

Reviewer #3: Yes

**********

PLOS authors have the option to publish the peer review history of their article (what does this mean?). If published, this will include your full peer review and any attached files.

If you choose “no”, your identity will remain anonymous but your review may still be made public.

**Do you want your identity to be public for this peer review?**  For information about this choice, including consent withdrawal, please see our Privacy Policy.

Reviewer #1: **Yes:**  Gordon A Dale

Reviewer #2: No

Reviewer #3: **Yes:**  Indrani Choudhuri

[NOTE: If reviewer comments were submitted as an attachment file, they will be attached to this email and accessible via the submission site. Please log into your account, locate the manuscript record, and check for the action link "View Attachments". If this link does not appear, there are no attachment files.]

**Figure resubmission:**

After uploading your figures to PLOS’s NAAS tool - https://ngplosjournals.pagemajik.ai/artanalysis, NAAS will process the files provided and display the results in the "Uploaded Files" section of the page as the processing is complete. If the uploaded figures meet our requirements (or NAAS is able to fix the files to meet our requirements), the figure will be marked as "fixed" above. If NAAS is unable to fix the files, a red "failed" label will appear above. When NAAS has confirmed that the figure files meet our requirements, please download the file via the download option, and include these NAAS processed figure files when submitting your revised manuscript.

**Reproducibility:**

?>

# Author comment: Author response to Decision Letter 1

**DOI:** 10.1371/journal.pcbi.1014859.r002

16 Jun 2026

Attachment Submitted filename: TCZ-BCZ Revision (PLoS Comp Bio).pdf

# Peer review: Decision Letter 1

**DOI:** 10.1371/journal.pcbi.1014859.r003

**Authors:** James Faeder, Philipp Altrock

28 Jul 2026

PCOMPBIOL-D-25-02726R1

How compartmentalization and proliferation shape the splenic T cell repertoire during an immune response to a complex antigen in mice

PLOS Computational Biology

Dear Dr. Textor,

Thank you for submitting your manuscript to PLOS Computational Biology. After careful consideration, we feel that it has merit but does not fully meet PLOS Computational Biology's publication criteria as it currently stands. Therefore, we invite you to submit a revised version of the manuscript that addresses the points raised during the review process.

Please submit your revised manuscript by Sep 27 2026 11:59PM. If you will need more time than this to complete your revisions, please reply to this message or contact the journal office at ploscompbiol@plos.org. When you're ready to submit your revision, log on to https://www.editorialmanager.com/pcompbiol/ and select the 'Submissions Needing Revision' folder to locate your manuscript file.

Please include the following items when submitting your revised manuscript:

* A letter that responds to each point raised by the editor and reviewer(s). You should upload this letter as a separate file labeled 'Response to Reviewers'. This file does not need to include responses to formatting updates and technical items listed in the 'Journal Requirements' section below.

* A marked-up copy of your manuscript that highlights changes made to the original version. You should upload this as a separate file labeled 'Revised Manuscript with Track Changes'.

* An unmarked version of your revised paper without tracked changes. You should upload this as a separate file labeled 'Manuscript'.

If you would like to make changes to your financial disclosure, competing interests statement, or data availability statement, please make these updates within the submission form at the time of resubmission. Guidelines for resubmitting your figure files are available below the reviewer comments at the end of this letter.

As the corresponding author, your ORCID iD is verified in the submission system and will appear in the published article. PLOS supports the use of ORCID, and we encourage all coauthors to register for an ORCID iD and use it as well. Please encourage your coauthors to verify their ORCID iD within the submission system before final acceptance, as unverified ORCID iDs will not appear in the published article. Only the individual author can complete the verification step; PLOS staff cannot verify ORCID iDs on behalf of authors.

We look forward to receiving your revised manuscript.

Kind regards,

Philipp Martin Altrock, Ph.D.

Academic Editor

PLOS Computational Biology

James Faeder

Section Editor

PLOS Computational Biology

**Additional Editor Comments (if provided):**

**Reviewers' comments:**

Reviewer's Responses to Questions

**Comments to the Authors:**

**Please note here if the review is uploaded as an attachment.**

Reviewer #1: The revised manuscript by Buytenhuijs et al. has adequately addressed all raised concerns. In its current form, I recommend the paper be accepted.

Reviewer #2: The revisions performed by the authors have improved and clarified the open points within the manuscript. All my previous points have been successfully addressed, except for a subpart in major comment 1 which is highlighted in yellow in the point-by-point reply to still be open (i.e., connection of time points)? However, I think this should not be a major issue and could be easily addressed/discussed in the discussion as maybe already planned.

**********

**Have the authors made all data and (if applicable) computational code underlying the findings in their manuscript fully available?**

The PLOS Data policy requires authors to make all data and code underlying the findings described in their manuscript fully available without restriction, with rare exception (please refer to the Data Availability Statement in the manuscript PDF file). The data and code should be provided as part of the manuscript or its supporting information, or deposited to a public repository. For example, in addition to summary statistics, the data points behind means, medians and variance measures should be available. If there are restrictions on publicly sharing data or code —e.g. participant privacy or use of data from a third party—those must be specified.

Reviewer #1: Yes

Reviewer #2: None

**********

PLOS authors have the option to publish the peer review history of their article (what does this mean?). If published, this will include your full peer review and any attached files.

If you choose “no”, your identity will remain anonymous but your review may still be made public.

**Do you want your identity to be public for this peer review?**  For information about this choice, including consent withdrawal, please see our Privacy Policy.

Reviewer #1: **Yes:**  Gordon A Dale

Reviewer #2: No

[NOTE: If reviewer comments were submitted as an attachment file, they will be attached to this email and accessible via the submission site. Please log into your account, locate the manuscript record, and check for the action link "View Attachments". If this link does not appear, there are no attachment files.]

**Figure resubmission:**

**Reproducibility:**

To enhance the reproducibility of your results, we recommend that authors of applicable studies deposit laboratory protocols in protocols.io, where a protocol can be assigned its own identifier (DOI) such that it can be cited independently in the future. Additionally, PLOS ONE offers an option to publish peer-reviewed clinical study protocols. Read more information on sharing protocols at https://plos.org/protocols?utm_medium=editorial-email&utm_source=authorletters&utm_campaign=protocols

# Author comment: Author response to Decision Letter 2

**DOI:** 10.1371/journal.pcbi.1014859.r004

22 Sep 2026

Attachment Submitted filename: TCZ-BCZ Revision (PLoS Comp Bio) (1).pdf

# Peer review: Decision Letter 2

**DOI:** 10.1371/journal.pcbi.1014859.r005

**Authors:** James Faeder, Philipp Altrock

27 Sep 2026

Dear Dr. Textor,

We are pleased to inform you that your manuscript 'How compartmentalization and proliferation shape the splenic T cell repertoire during an immune response to a complex antigen in mice' has been provisionally accepted for publication in PLOS Computational Biology.

Before your manuscript can be formally accepted you will need to complete some formatting changes, which you will receive in a follow up email. A member of our team will be in touch with a set of requests.

Please note that your manuscript will not be scheduled for publication until you have made the required changes, so a swift response is appreciated.

IMPORTANT: The editorial review process is now complete. PLOS will only permit corrections to spelling, formatting or significant scientific errors from this point onwards. Requests for major changes, or any which affect the scientific understanding of your work, will cause delays to the publication date of your manuscript.

Should you, your institution's press office or the journal office choose to press release your paper, you will automatically be opted out of early publication. We ask that you notify us now if you or your institution is planning to press release the article. All press must be co-ordinated with PLOS.

Thank you again for supporting Open Access publishing; we are looking forward to publishing your work in PLOS Computational Biology.

Best regards,

Philipp Martin Altrock, Ph.D.

Academic Editor

PLOS Computational Biology

James Faeder

Section Editor

PLOS Computational Biology

***********************************************************

Reviewer's Responses to Questions

**Comments to the Authors:**

**Please note here if the review is uploaded as an attachment.**

Reviewer #2: All my previous comments were appropriately addressed by the authors. I have no further comments.

**********

**Have the authors made all data and (if applicable) computational code underlying the findings in their manuscript fully available?**

The PLOS Data policy requires authors to make all data and code underlying the findings described in their manuscript fully available without restriction, with rare exception (please refer to the Data Availability Statement in the manuscript PDF file). The data and code should be provided as part of the manuscript or its supporting information, or deposited to a public repository. For example, in addition to summary statistics, the data points behind means, medians and variance measures should be available. If there are restrictions on publicly sharing data or code —e.g. participant privacy or use of data from a third party—those must be specified.

Reviewer #2: None

**********

PLOS authors have the option to publish the peer review history of their article (what does this mean?). If published, this will include your full peer review and any attached files.

If you choose “no”, your identity will remain anonymous but your review may still be made public.

**Do you want your identity to be public for this peer review?**  For information about this choice, including consent withdrawal, please see our Privacy Policy.

Reviewer #2: No

# Editor report: Acceptance letter

**DOI:** 10.1371/journal.pcbi.1014859.r006

**Authors:** James Faeder, Philipp Altrock

PCOMPBIOL-D-25-02726R2

How compartmentalization and proliferation shape the splenic T cell repertoire during an immune response to a complex antigen in mice

Dear Dr Textor,

I am pleased to inform you that your manuscript has been formally accepted for publication in PLOS Computational Biology. Your manuscript is now with our production department and you will be notified of the publication date in due course.

The corresponding author will soon be receiving a typeset proof for review, to ensure errors have not been introduced during production. Please review the PDF proof of your manuscript carefully, as this is the last chance to correct any errors. Please note that major changes, or those which affect the scientific understanding of the work, will likely cause delays to the publication date of your manuscript.

Soon after your final files are uploaded, unless you have opted out, the early version of your manuscript will be published online. The date of the early version will be your article's publication date. The final article will be published to the same URL, and all versions of the paper will be accessible to readers.

For Research, Software, and Methods articles, you will receive an invoice from PLOS for your publication fee after your manuscript has reached the completed accept phase. If you receive an email requesting payment before acceptance or for any other service, this may be a phishing scheme. Learn how to identify phishing emails and protect your accounts at https://explore.plos.org/phishing.

Thank you again for supporting PLOS Computational Biology and open-access publishing. We are looking forward to publishing your work!

With kind regards,

Anitha Samidurai

PLOS Computational Biology | Carlyle House, Carlyle Road, Cambridge CB4 3DN | United Kingdom ploscompbiol@plos.org | Phone +44 (0) 1223-442824 | ploscompbiol.org | @PLOSCompBiol
